# Proteome-Wide Investigation of Proline Hydroxylation in Pancreatic Ductal Adenocarcinoma Using DiLeu Isobaric Labeling Strategy

**DOI:** 10.1016/j.mcpro.2025.100969

**Published:** 2025-04-09

**Authors:** Feixuan Wu, Dylan Nicholas Tabang, Danqing Wang, Jon S. Odorico, Lingjun Li

**Affiliations:** 1 School of Pharmacy, University of Wisconsin-Madison, Madison, Wisconsin, USA; 2 Department of Chemistry, University of Wisconsin-Madison, Madison, Wisconsin, USA; 3 Department of Pathology, Boston Children's Hospital & Harvard Medical School, Boston, Massachusetts, USA; 4 Division of Transplantation, Department of Surgery, School of Medicine and Public Health, University of Wisconsin-Madison, Madison, Wisconsin, USA; 5 Biophysics Graduate Program, University of Wisconsin-Madison, Wisconsin, USA; 6 Lachman Institute for Pharmaceutical Development, School of Pharmacy, University of Wisconsin-Madison, Madison, Wisconsin, USA; 7 Wisconsin Center for NanoBioSystems, School of Pharmacy, University of Wisconsin-Madison, Madison, Wisconsin, USA

**Keywords:** hydroxyproline, pancreatic ductal adenocarcinoma (PDAC), isobaric labeling for quantitation, DiLeu tagging, HILIC

## Abstract

Pancreatic ductal adenocarcinoma (PDAC) is a highly aggressive malignancy characterized by a dense fibrotic stroma intertwined with a collagen-rich extracellular matrix (ECM), which significantly contributes to tumor progression. In this study, we developed a high-throughput quantitative method that integrates enhanced hydrophilic interaction liquid chromatography (HILIC) with modified elution conditions and 12-plex *N,N*-dimethyl leucine (DiLeu) isobaric tags, facilitating efficient multiplexed quantitative analysis of hydroxyproline. This approach was applied to human pancreatic samples and resulted in the identification of 194 hydroxyproline peptides from 157 hydroxyproline sites and 59 proline-hydroxylated proteins, representing the first and the largest hydroxyproline proteomics dataset reported for the pancreas to date. This dataset lays a molecular foundation for understanding the structure-function relationships of hydroxyproline-containing proteins and their roles in pancreatic physiology and pathology. We then apply this strategy to investigating proline hydroxylation alterations in benign pancreatic tumors, PDAC, and their normal adjacent tissues (NAT). Our findings suggest significant biological functions related to proline hydroxylation, including altered patterns of key proteins such as collagen alpha-1(I) chain and collagen alpha-1(XII) chain. These proteins emerge as potential targets for further studies on proline hydroxylation in PDAC, potentially elucidating its role in modifying protein structures and influencing cancer progression.

## Highlights

• High-throughput quantitative method for proline hydroxylation analysis.
• Integration of S-Trap, isobaric tags, and HILIC for sample preparation.
• Comprehensive characterization of hydroxyproline alterations during PDAC progression.
• Identification of potential proline hydroxylation targets in PDAC.

## Graphical Abstract

**Figure:**
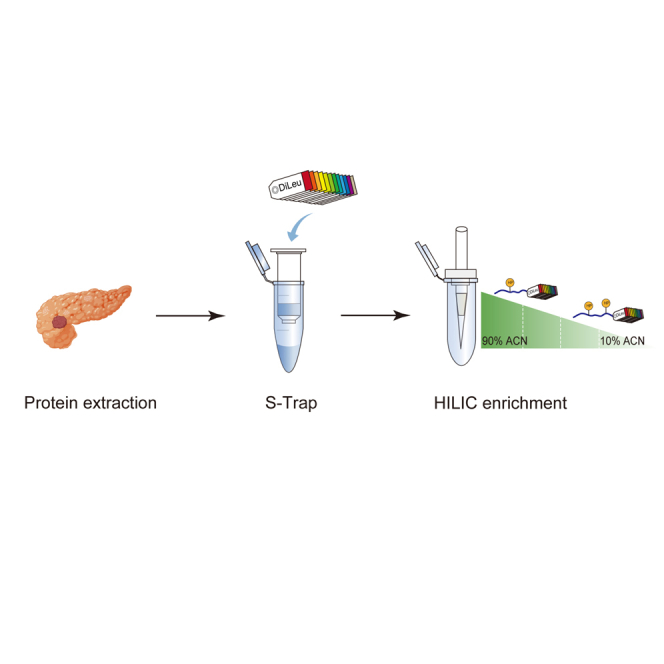

## In Brief

We present an exploratory investigation into proline hydroxylation as a potential biomarker, mapping its landscape and alterations in hydroxyproline across benign and malignant pancreatic ductal adenocarcinoma (PDAC) tumors as well as adjacent normal tissues. Our workflow integrates S-Trap, DiLeu isobaric labeling, and hydrophilic interaction liquid chromatography (HILIC) into a desalting-free, high-throughput approach. This analysis will shed light on the hydroxyproline profile, highlighting key differences in proline hydroxylation patterns, and identify noteworthy hydroxylated-proline-containing protein candidates during pancreatic cancer progression.

With a 5-year survival rate below 10%, PDAC ranks among the most lethal solid malignancies and is expected to become the second-leading cause of cancer-related deaths by 2030 (1, 2). The lack of early symptoms and effective screening methods results in up to 85% of patients being diagnosed with locally advanced or unresectable metastatic disease (3, 4). First-line treatment for metastatic PDAC typically involves combination cytotoxic chemotherapy, which only modestly extends survival (5). For patients with metastatic disease, the median overall survival remains less than 12 months (6).

Despite these treatments, PDAC continues to exhibit significant drug resistance, leading to extremely poor outcomes. Contributing factors to this resistance include the dense fibrous stroma surrounding the tumor, the abnormal vasculature network, and the immune-suppressive microenvironment characteristic of this cancer type (3). The tumor microenvironment in PDAC consists of a rigid ECM. This ECM stiffness and desmoplasia not only provide structural support but also create a specific microenvironment that promotes tumor growth, metastasis, and survival, and acts as a barrier to chemotherapeutic drugs (7, 8, 9). The ECM is composed of collagen, elastin, fibronectin, hyaluronan, and other glycosaminoglycans, forming a dense network alongside fibroblasts, endothelial cells, and infiltrating immune cells. Specifically, collagen makes up a significant portion of the ECM in PDAC, often constituting around 60% to 80% of the total protein content in the ECM (10). Up to 40% of collagen carries hydroxyproline, making proline hydroxylation one of the most abundant post-translational modifications (PTMs) (11). Proline hydroxylation occurs at the Cγ atom, producing hydroxyproline by an enzymatic reaction of prolyl hydroxylase (12). This modification stabilizes the triple helix structure, an essential feature of collagen protofibrils (11). It also plays a key role in signaling, particularly in oxygen-sensing pathways, angiogenesis, and tumor cell proliferation (13). Nevertheless, there are few reports on the characterization of proline hydroxylation in PDAC (14, 15, 16). This is largely due to the lack of methods to enrich the hydroxyproline changes in complex biological samples. Therefore, the development of a method to detect the changes in proline hydroxylation in both PDAC biological samples and on a large scale would significantly enhance our understanding of the pathological mechanisms of PDAC.

Available approaches for proline hydroxylation analysis include antibody-based enrichment and chemical derivatization (17, 18). Despite tremendous advances in these strategies, these methods are either limited in availability or need additional cleanup steps. HILIC is typically known as a useful technique to enrich both *N*- and *O*-glycopeptides in complex biological samples on a large scale, and has found successful applications in various systems, such as biofluids and tissues (19, 20, 21). McNulty *et al* reported HILIC marks a significant advance in phosphoproteomics by leveraging the strong hydrophilicity of phosphate groups to selectively enrich and fractionate phosphopeptides (22). Given the additional hydrophilicity afforded by the hydroxyl group in hydroxyproline, the potential of HILIC for the enrichment of a diverse set of hydrophilic peptides, including hydroxyproline peptides, remains underexplored. Furthermore, dissecting the involvement of proline hydroxylation in the onset and progression of PDAC necessitates comparative analysis. However, even fewer studies were able to provide quantitative information to our knowledge. To enable high-throughput quantitative proteomic analysis in a single MS/MS run, stable isotope labeling techniques, particularly isobaric labeling methods such as commercially available tandem mass tags (TMTs) (23, 24) and custom-developed DiLeu isobaric tags (25), have gained popularity for comparative studies of different biological states. These approaches have demonstrated their effectiveness in producing accurate quantitative results with high multiplexing capability, reduced run-to-run variations, enhanced analytical throughput in numerous proteomics studies (26, 27, 28) and PTM analyses (20, 29, 30). Compared to commercially available tags, DiLeu tags are considerably more cost-effective and can be synthesized in-house with high yields (25). Here, we hypothesize that the combination of DiLeu isobaric labeling with the HILIC fractionation method will enable high-throughput quantitation of proline hydroxylation.

In the present study, we develop a global quantification strategy enabling fractionation of hydroxyproline peptides labeled with 12-plex DiLeu isobaric tags and apply this pipeline to investigate proline hydroxylation alterations in various types of PDAC. This work lays a foundation for a more in-depth investigation into the functional roles of these proline-hydroxylated proteins in pancreatic cancer progression.

## Experimental Procedures

### Chemicals and Materials

Dithiothreitol (DTT), PNGase F, and sequencing grade trypsin were from Promega (Madison, WI). Optima liquid chromatography/mass spectrometry (LC/MS) grade solvents, formic acid (FA), urea, sodium chloride, phosphoric acid, ammonium hydroxide, and sodium carbonate were from Fisher Scientific (Pittsburgh, PA). Acetonitrile (ACN), trifluoroacetic acid (TFA), iodoacetamide (IAA), triethylammonium bicarbonate (TEAB), *N,N*-dimethylformamide (DMF), 4-(4,6-dimethoxy-1,3,5-triazin-2-yl)-4-methylmorpholinium tetrafluoroborate (DMTMM) were purchased from Sigma-Aldrich. Suspension trapping (S-Trap) was purchased from Protifi. C18 SepPak cartridges were purchased from Waters Corporation. Empty 200 *μ*l TopTips were from Glygen Corp. Strong cation exchange (SCX) spin tips were purchased from PolyLC. Poly HYDROXYETHYL A bulk material was purchased from PolyLC. Protease inhibitor cocktail tablets and phosphatase inhibitor cocktail tablets were from Roche. All other chemicals and LC-MS grade solvents were purchased from Fisher Scientific.

### Specimen Acquisition

Human pancreas tissues were procured by the University of Wisconsin Organ and Tissue Donation Services from donors with no indication of diabetes or pancreatitis, with consent obtained for research from next of kin. Following organ recovery, pancreata were allocated for research if deemed unfit for transplantation due to vascular damage during organ recovery, no suitable recipient, and nonideal age or body mass index. The organs were received within 24 h of recovery and trimmed of extra-pancreatic connective tissues, including duodenum, large arteries, and veins. The parenchyma was cut into 1 cm^3^ cubes and frozen at −80 °C for future use. Some pieces were also immediately fixed with 4% paraformaldehyde for histology. One piece of frozen pancreas per donor was thawed and rinsed with 1 × phosphate-buffered saline, followed by sterile water, and then manually chopped into small pieces. The pieces were immersed in sterile water and homogenized for 3 s, then pelleted (16,100*g*, 5 min). Any floating lipids were removed, and the translucent supernatant was discarded. The pellet was flash frozen and stored at −80 °C. Donor information can be found in supplemental Table S1.

The tumors and NAT were obtained without protected health information from the TSB BioBank, which maintains an IRB-approved protocol (UW HS IRB# 2016-0934) and an honest broker system for the collection and distribution of samples from consented subjects, meeting the definition of nonhuman subject research. The acquisition procedures were the same as described above. Part of benign tumors and their NAT were embedded in optimal cutting temperature (OCT) compound for processing as previously reported (31). The details are included in supplemental Table S2.

### Sample Preparation

The tissue samples were homogenized in SDS lysis buffer composed of 5% SDS, 5 mM CaCl_2_, 20 mM NaCl, and 50 mM TEAB, with one protease inhibitor tablet and one phosphatase inhibitor tablet added per 10 ml of lysis buffer. The lysate was then subjected to sonication using a probe sonicator in an ice-water bath at 50% power, with 5-s on/off pulses for three cycles. Following sonication, the lysates were centrifuged at 14,000*g* for 15 min at 4 °C, and the resulting supernatant containing proteins was collected and quantified using a BCA assay. The proteins were reduced with 5 mM DTT at 37 °C for 1 h, followed by alkylation with 15 mM IAA at room temperature in the dark for 30 min. The alkylation reaction was subsequently quenched by the addition of 5 mM DTT for 10 min. Finally, additional SDS was added to achieve a final concentration of 5%. Samples were then prepared using S-Trap according to the manufacturer’s instructions. For the PNGase F treatment group, *N*-glycans were removed by incubating with PNGase F (enzyme-to-protein ratio of 1:20) at 37 °C for 1 h. Trypsin/LysC was then added (enzyme-to-protein ratio of 1:10) and incubated at 47 °C for 2 h. Eluted peptides were dried down in SpeedVac.

### DiLeu Labeling

The synthesis and labeling of DiLeu tags were conducted, as previously reported (25). Briefly, DiLeu tags were activated in anhydrous DMF with DMTMM and NMM at 0.6 × molar ratios to tags. The mixture was vortexed at room temperature for 1 h, and the supernatant was then added to each for labeling. After vortexing at room temperature for 2 h, the reaction was quenched by adding 5% NH_2_OH to a final concentration of 0.25%. Each batch of labeled peptides were pooled together and dried down *in vacuo*. Aliquots of labeled peptides were cleaned with SCX spin tips according to the manufacturer’s protocols and then desalted with C18 SepPak cartridges. Desalted peptides were dried *in vacuo*.

### High pH Fractionation

High pH (HpH) fractionation was performed on a Waters Alliance e2695HPLC using a C18 reversed-phase column (2.1 mm × 150 mm 5 μm, 100 Å, PolyLC) operating at 0.2 ml/min. Mobile phase A consisted of 10 mM ammonium formate at pH 10 adjusted with ammonium hydroxide, and mobile phase B consisted of 90% ACN and 10 mM ammonium formate at pH 10. Separation was achieved with a gradient as following: 1% B (0–5 min), 1 to 40% B (5–50 min), 40 to 60% B (50–54 min), 60 to 70% B (54–58 min), and 70 to 100% B (58–59 min). Fractions were collected every 4 min, and nonadjacent fractions were concatenated into four samples before being dried *in vacuo* for LC-MS/MS analysis.

### HILIC Fractionation

Fractionation of DiLeu-labeled hydroxyproline peptides was performed with in-house-packed HILIC SPE tips. 3 mg of cotton wool was plugged into a 200 *μ*l empty TopTip, which was placed on a 2 ml microcentrifuge tube with an adapter unit. After activation in 1% TFA for 15 min, the material was transferred into the cotton-packed TopTip at a beads-to-peptide ratio of 30:1. The solvent was removed at 200*g* for 2 min. The stationary phase was conditioned with 300 *μ*l of 1% TFA and loading buffer (95% ACN/1% TFA) 3 times. 700 *μ*g of DiLeu-labeled sample was dissolved in 300 *μ*l of loading buffer and loaded onto the HILIC-cotton tip. The tip was centrifuged at 200*g* for 2 min, and the flow-through was reloaded to the HILIC-cotton four or five times to ensure complete retention. The HILIC-cotton tip was washed with 300 *μ*l of loading buffer 6 times. For pooled pancreas samples, five fractions were collected: (1) 300 *μ*l of 90% ACN/5% FA, (2) 300 *μ*l 80% ACN/5% FA, (3) 300 *μ*l 70% ACN/5% FA, (4) 300 *μ*l 50% ACN/5% FA, and (5) 300 *μ*l 10% ACN/5% FA. For tumors and NAT samples, seven fractions were collected for analysis: (1) 300 *μ*l 90% ACN/5% FA, (2) 300 *μ*l 85% ACN/5% FA, ((3) 300 *μ*l 80% ACN/5% FA, (4) 300 *μ*l 70% ACN/5% FA, (5) 300 *μ*l 60% ACN/5% FA, (6) 300 *μ*l 50% ACN/5% FA, and (7) 300 *μ*l 10% ACN/5% FA. Samples were dried down *in vacuo* before MS analysis.

### NanoLC-MS/MS Analysis

Human pooled pancreas samples were analyzed using a Q Exactive Orbitrap mass spectrometer (Thermo Fisher Scientific). Samples were reconstituted in 0.1% FA and separated on a 15 cm column packed with Ethylene Bridge Hybrid C18 material (1.7 μm, 130 Å; Waters). The mobile phase consisted of water with 0.1% FA as phase A and ACN with 0.1% FA as phase B. Chromatographic separation was achieved through gradient elution, with mobile phase B increasing from 3% to 30% over 100 min at a flow rate of 0.3 *μ*l/min. Full MS data was acquired across a mass scan range of *m/z* 300 to 1500 at a resolution of 70,000, with an automatic gain control (AGC) target of 1 × 10^6^ and a maximum injection time (IT) of 250 ms. For data-dependent acquisition (DDA) tandem mass spectrometry, the top 15 precursor ions were selected for MS2 analysis using higher-energy collisional dissociation (HCD) with a resolution of 70,000, an AGC target of 1 × 10^5^, a maximum IT of 150 ms, an isolation width of *m/z* 1.4, a fixed first mass of *m/z* 110, a normalized collision energy (NCE) of 30, and a dynamic exclusion duration of 20 s. Each sample was analyzed in technical replicates.

The analysis of human PDAC tumors, benign tumors, and their NAT was performed using a Fusion Lumos mass spectrometer (Thermo Fisher Scientific) coupled with a Dionex Ultimate 3000 UPLC system (Thermo Fisher Scientific). Samples were reconstituted in 0.1% FA and separated on a 15 cm in-house packed BEH C18 capillary column (1.7 μm, 130 Å; Waters) using a gradient from 3% to 40% ACN in 0.1% FA over 100 min, at a flow rate of 0.3 μl/min. For hydroxyproline peptide analysis, MS scans were acquired from *m/z* 300 to 1800 at a resolution of 60,000, with an AGC target of 2 × 10^5^ and an IT of 50 ms. MS/MS spectra were obtained in a DDA mode, selecting the top 20 precursors for fragmentation using a NCE of 30. The MS/MS settings included a resolution of 60,000, an AGC target of 5 × 10^4^, and a maximum IT of 118 ms. For the proteomics analysis, survey scans of peptide precursors were conducted from *m/z* 300 to 1800 at a resolving power of 60,000, with an AGC target of 1 × 10^5^ and a maximum IT of 100 ms. The MS/MS spectra were also acquired in a top 20 DDA mode with a fixed NCE of 30, a resolution of 60,000, an AGC target of 8 × 10^4^, and a maximum IT of 100 ms. Precursors were subjected to dynamic exclusion for 15 s with a tolerance of 10 ppm. Each sample was acquired in technical replicates.

### Data Analysis

Hydroxyproline peptide and protein data were processed using MaxQuant (version 1.5.2.8). The raw files were searched against the UniProt *Homo sapiens* reviewed database (20,367 sequences, December 2023). Trypsin and LysC were specified as enzymes, allowing for a maximum of two missed cleavages. Mass tolerance for precursor ions was 4.5 ppm and the fragment ion mass tolerance was 20 ppm. Quantification was based on reporter ion during MS2 analysis, with DiLeu labeling assigned to peptide N-termini and lysine residues as isobaric labels. The reporter mass tolerance was set to 0.01 Da. Carbamidomethylation (+57.02146 Da) on cysteine residues and 12-plex DiLeu (+145.12801 Da) on peptide N-terminus and lysine residues were defined as fixed modifications. Dynamic modifications included methionine oxidation (+15.99492 Da), protein N-termini acetylation (+42.01056 Da), and proline hydroxylation (+15.99492 Da). A maximum of five modifications per peptide was allowed. Proteomics data were analyzed similarly, except without the dynamic modification of proline hydroxylation. The search results were filtered with a 1% false discovery rate (FDR) at both the peptide and protein levels. Reverse hits and potential contaminants were excluded, and the proline hydroxylation site localization probability threshold was set at 0.75. MS/MS spectra of hydroxyproline peptide PSMs were manually inspected to ascertain the accurate assignment. All other parameters were kept at their default settings. Missing intensity values were imputed using the "replace missing values from normal distribution" function in Perseus (version 2.0.11.0). Statistical analysis was performed using a paired two-sample Student’s *t* test with a two-tailed distribution for binary comparisons and one-way ANOVA, with *p*-values adjusted using permutation-based FDR for multiple testing corrections. Bioinformatics analyses including hierarchical clustering, volcano plots, and gene network plot are using Hiplot (https://hiplot.com.cn/) and Python programing. Pathway enrichment analysis was conducted through WebGestalt (https://www.webgestalt.org/).

### Experimental Design and Statistical Rationale

Firstly, for method development utilizing pooled healthy pancreas tissues, hydroxyproline peptide enrichment methods were evaluated to optimize strategy and maximize the hydroxyproline proteome coverage. Commonly used methods, including HILIC and HpH fractionation with or without PNGase F treatment, were evaluated, respectively. For human PDAC tumors and NAT hydroxyproline analysis, six pairs of malignant or benign tumors and their NAT were used. Trypsin/LysC digestion, DiLeu isobaric labeling, and following optimized HILIC strategies were applied to the pancreas samples. The global proteome dataset was used for normalization to account for variations in protein abundance. A total of seven fractions of enriched hydroxyproline peptides were collected. For each fraction, two technical replicates were performed for LC-MS/MS analysis. Statistical analyses are described in Data Analysis.

## Results

### In-Depth Hydroxyproline Quantification Using 12-Plex DiLeu Isobaric Tags

**Fig. 1:**
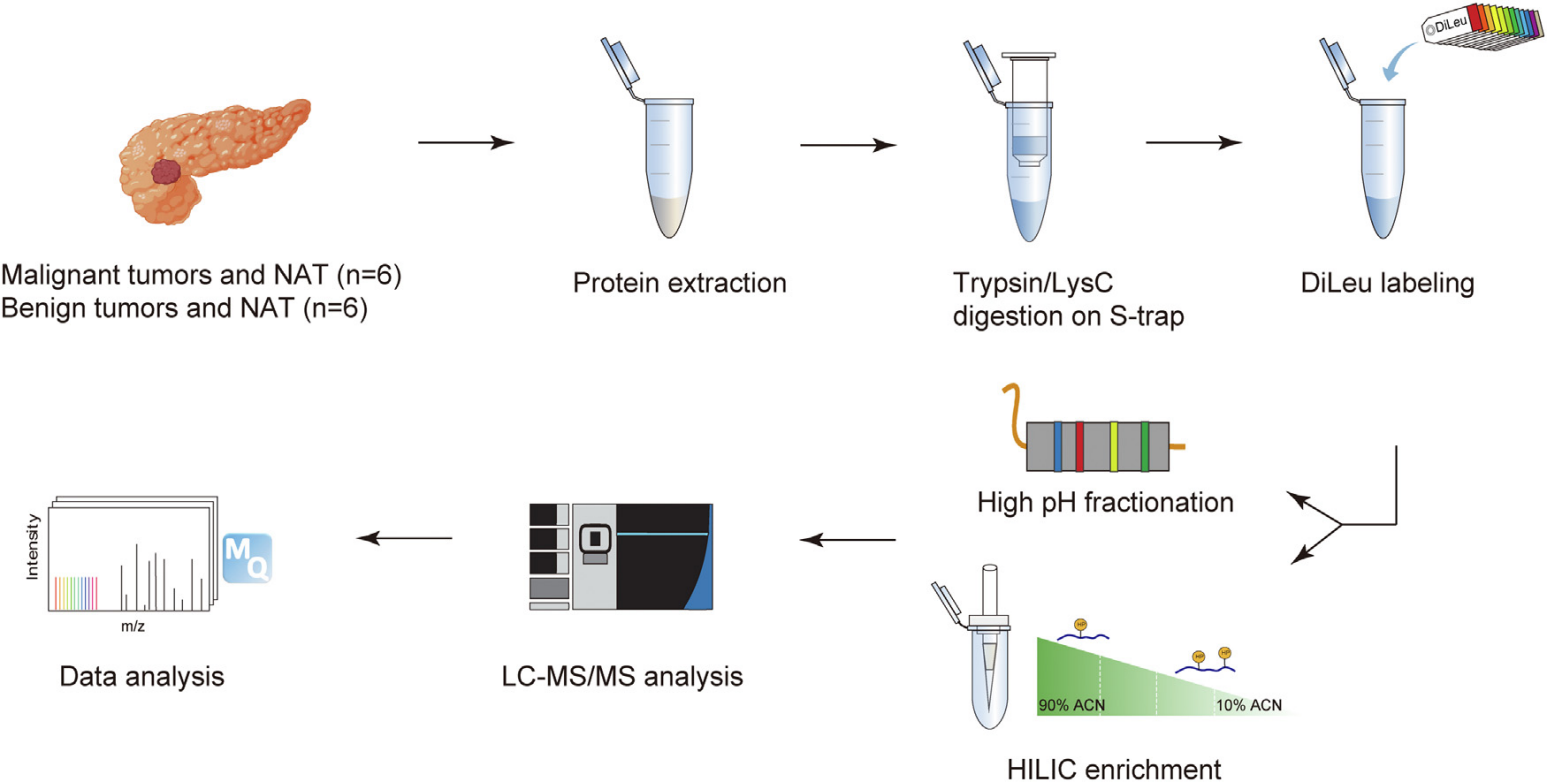
**Experimental workflow of quantitative analysis using 12-plex DiLeu isobaric labeling strategy**. Tissues were cryo-pulverized into powder, and protein was extracted using sonication. Contaminant removal and protein digestion were performed using S-Trap. 12-plex DiLeu isobaric labeling was used before HILIC enrichment to achieve quantitative hydroxyproline proteomics analysis. The enriched peptides were analyzed using LC-MS/MS, and data was processed with MaxQuant (figure created in part using Biorender.com).

We performed a one-tube quantitative analysis of malignant, benign tumors and their paired NATs (Fig. 1). The S-Trap method circumvents the necessity of sample desalting and allows for the preparation of SDS-containing protein lysates in a fraction of the time needed for filter-aided sample preparation (FASP) (32). It possesses the advantages of FASP and other filter-based methods while decreasing the sample handling steps and time. Also, it has been shown to outperform FASP and in-solution digestions with the greatest number of unique protein identifications (32).

Although advances in various analytical technologies have made large-scale analysis of hydroxyproline peptides feasible, the depth of hydroxyproline proteome study in a complex sample has been limited compared with other PTMs studies, such as glycoproteomics and phosphoproteomics (33, 34, 35, 36). This is because hydroxyproline only constitutes a minor portion of the total peptide mixtures and lacks an efficient enrichment method to enable successful analysis of hydroxyproline peptides in complex biological samples. Some studies performed HpH fractionation to improve the coverage (27, 29), while others used HILIC after removing *N*-glycans (37). HILIC enrichment is typically used for the enrichment of the intact *N*-glycopeptides. It can also be modified to enrich intact *O*-glycopeptides by increasing the percentage of organic solvents (38). On the other hand, off-line fractionation such as HpH fractionation is often utilized due to its ease of implementation and its compatibility with large amounts of starting material, which has also been shown to be highly orthogonal to the subsequent LC-MS/MS analysis with low-pH reversed-phase chromatography (39). We first compared the performances between HpH and HILIC using healthy pancreas tissues, and then applied the optimal method to the analyses of the PDAC samples to demonstrate the ability of our method to detect hydroxyproline peptides.

### Optimization of Sample Preparation

**Fig. 2:**
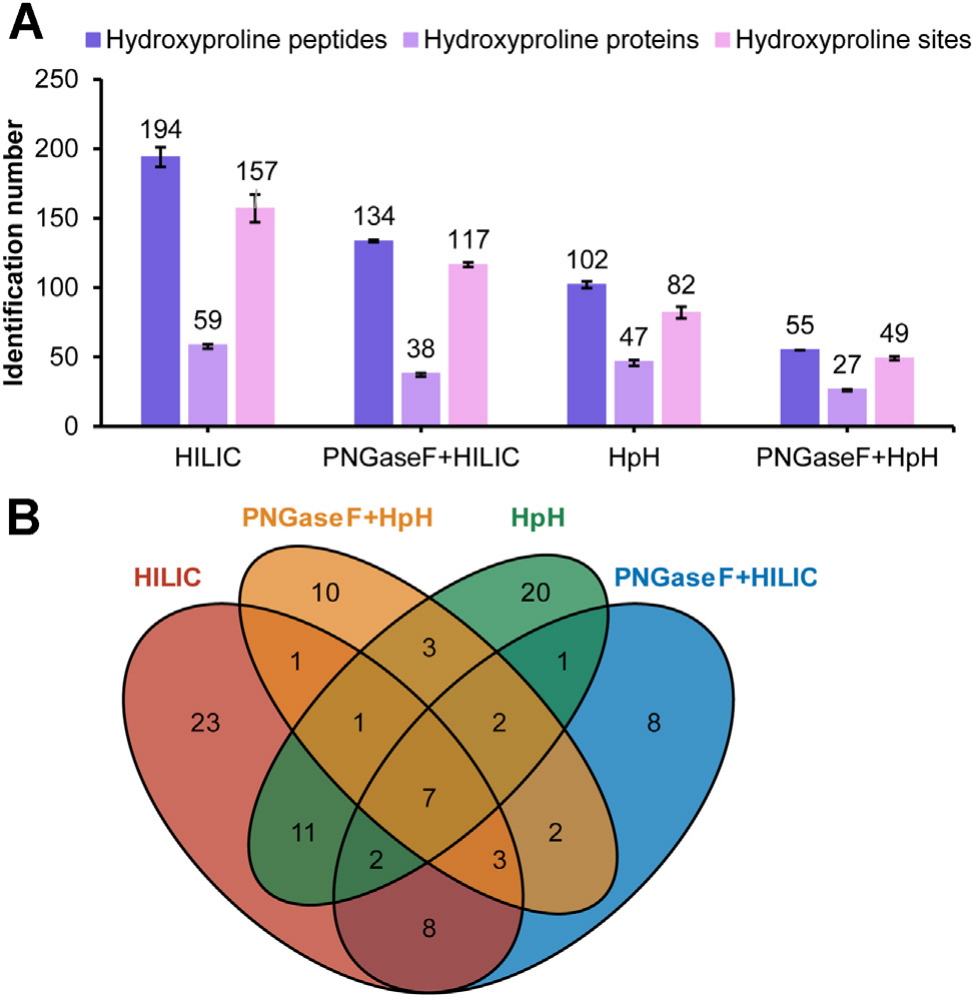
**Comparison of sample preparation using HILIC and HpH methods**. Different strategies, including HILIC and HpH with or without PNGase F treatment, were employed to enrich hydroxyproline peptides from pancreas tissues. The error bar represented sample standard deviation of two technical replicates. *A*, identification number in hydroxyproline analysis. *B*, overlap of hydroxyproline proteins by the four methods, as shown using the Venn diagram.

Here, we adapt and optimize this strategy to conduct desalting-free DiLeu labeling. To assess the performance of this optimized one-tube DiLeu labeling sample processing, the same amounts of pancreas tissue labeled with 3-plex DiLeu isobaric tags (116b, 117a and 118d) were processed with or without PNGase F treatment, followed by the HILIC method or HpH fractionation method. Unlike the conventional HILIC method, we increased the organic solvents in elution buffer from 10% ACN to 90% ACN, as hydroxyproline peptides are less hydrophilic than glycopeptides. For DiLeu-labeled hydroxyproline peptides, the one-tube processing workflow outperformed the HpH fractionation method in all aspects, including the identification of unique hydroxyproline peptides, and corresponding hydroxyproline proteins and hydroxyproline sites (Fig. 2*A*). The method with HILIC fractionation has a 2-fold increase of the number of hydroxyproline peptides and sites in the HpH fractionation method. Also, the proportion of hydroxyproline peptides relative to all peptides in the HILIC method is 6.4%, with values of 5.0%, 5.1%, 8.3%, 8.3%, and 5.8% across fractions E1 to E5, respectively. Compared with the method treated with PNGase F, PNGase F seems to cause loss of hydroxyproline peptides and leads to decrease of identifications in both HILIC and HpH methods. This might be due to peptide loss and change of hydrophilicity and binding properties after deglycosylation. Consistently, Figure 2*B* shows that the method without PNGase F treatment and HILIC fractionation has the most overlap of proline hydroxylated proteins with other methods (58.9%). Additionally, the widest range of GRAVY scores in HILIC method indicate the greatest diversity in the hydrophobicity profiles of the peptides (supplemental Fig. S1*A*). To achieve the best coverage of hydroxyproline peptides, the modified HILIC method was employed in the subsequent experiments for proline hydroxylated proteomics analysis in PDAC.

### Profiling and Quantification of Proline Hydroxylation in Healthy Pancreas

**Fig. 3:**
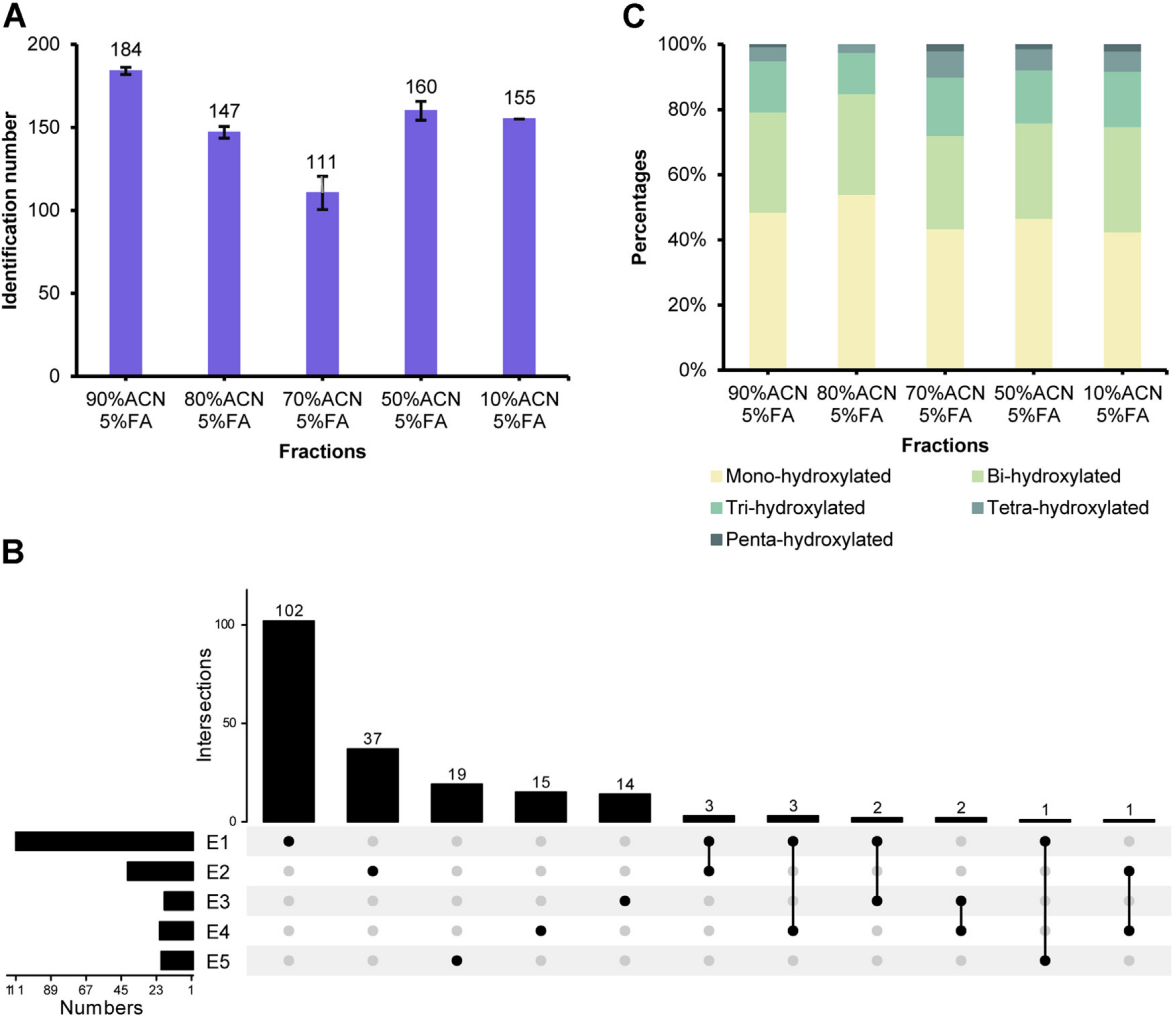
**Quantitative analysis of proline hydroxylation from human pancreas using the HILIC method**. *A*, the number of hydroxyproline peptides through each fraction. The error bar represented the sample standard deviation. *B*, upset plot of hydroxyproline peptides between each fraction. *C*, hydroxyproline peptide and multi-hydroxyproline peptide percentages in five fractions.

As discussed previously, the current depth of proline hydroxylation in the pancreas is far from being satisfactory, which will largely hinder hydroxyproline-based biomarker discovery studies in the pancreas for various diseases. Here, the established multiplexed hydroxyproline peptide fractionation method based on the HILIC material was applied to the in-depth proline hydroxylated proteome analysis of the pancreas sample. In this method, the highly acidic content of an organic solvent (95% ACN/1% TFA) was employed as the loading buffer, which could enhance the hydroxyproline peptide retention via hydrophilic interaction, while slightly lower acidic content of decreasing organic solvents was used as the elution buffer, which could release the hydroxyproline peptides (Fig. 3*A*). As shown in Figure 2*A*, 194 hydroxyproline peptides corresponding to 59 hydroxyproline proteins were identified by the HILIC method. Figure 3*B* displays the overlap of hydroxyproline peptides identified in five fractions, and 91.9%, 90.2%, 77.8%, 71.4%, and 95.0% of hydroxyproline peptides were unique in each fraction, indicating the high separation efficiency provided by the HILIC method. The number of hydroxyproline peptides reduced as the organic solvents decreased (Fig. 3, *A* and *B*). Interestingly, with lower ACN concentration, more multi-hydroxyl peptides were eluted in later fractions, which are usually underexplored in conventional HILIC enrichment (37). The modified elution buffers exhibited better retention of these peptides as more of them were distributed in the last three fractions (Fig. 3*C*). Moreover, E1 has the largest range of GRAVY scores (supplemental Fig. S1*B*), which is consistent with the most identified IDs. Among the identified proline hydroxylated proteins, 162 out of 225 are ECM proteins and thus show high efficiency of this method (supplemental Table S3). These results highlight the effectiveness of the method and are consistent with previous reports that proline hydroxylation contributes to the structural and functional integrity of ECM (40, 41).

### Alterations in Proline Hydroxylation during PDAC Progression

ECM is well-recognized as a critical factor in pancreatic cancer development and progression, and understanding the roles of hydroxyproline can facilitate studies in pancreatic cancer pathogenesis and targeted therapeutic approaches (42). Previous studies have shown that cancer progression induces dysregulated collagen (7, 10) but few studies report on hydroxyproline due to the limitations in analytical tools. Here, we adapt our method using 12-plex DiLeu tags to quantitatively investigate how protein proline hydroxylation changes in pancreatic cancer progression in a larger-scale manner and aim to provide deeper insights into biological significance.

**Fig. 4:**
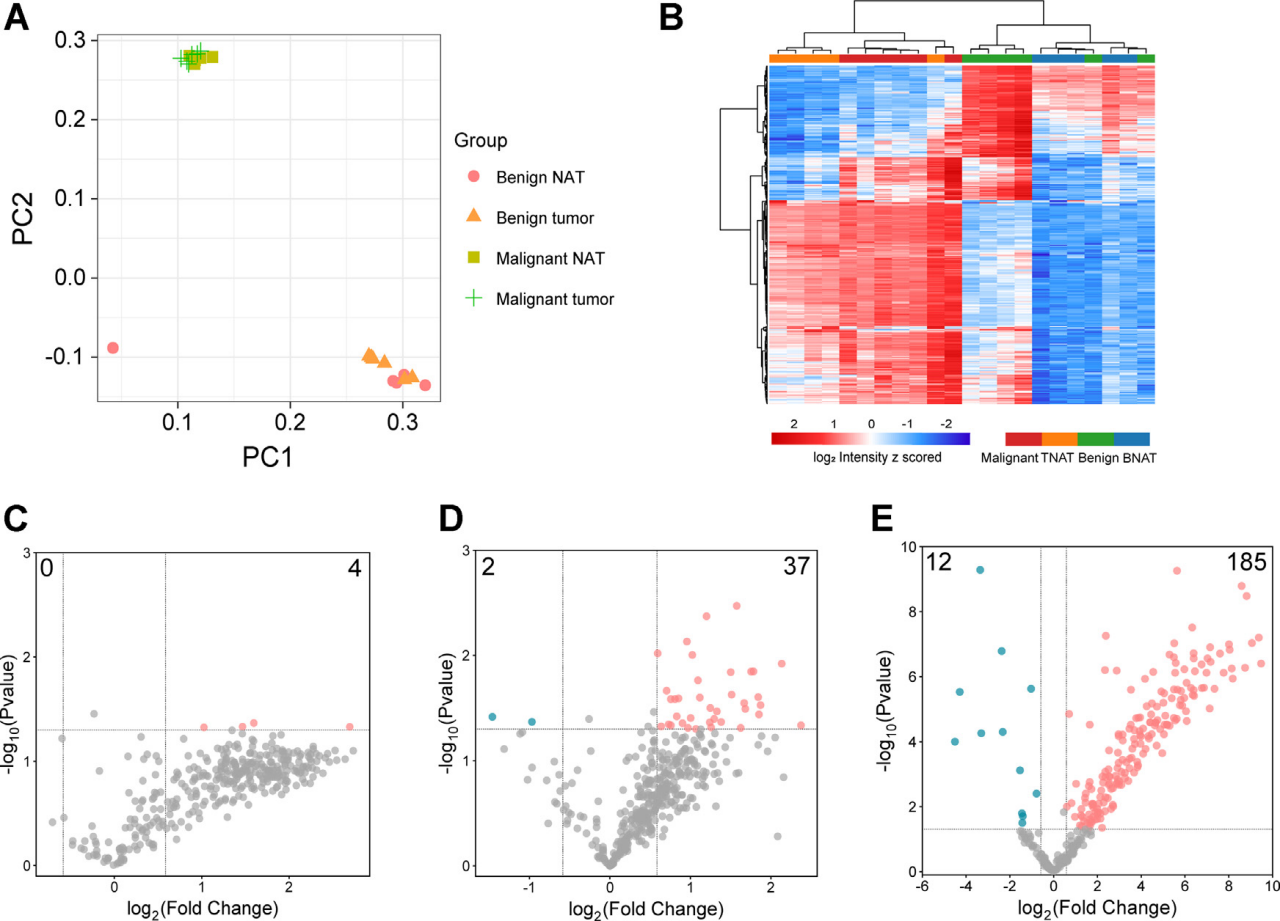
**Quantitative analysis of proline hydroxylation in pancreatic tissues and across different groups.***A*, PCA plots at proline hydroxylation levels of benign tumors, their NAT, malignant tumors, and their NAT. *B*, hierarchical clustering analysis of DiLeu reporter ion intensities of all quantified hydroxyproline peptides in NAT and tumor groups. Volcano plots show pairwise comparisons of hydroxyproline peptide expression levels between benign tumors and their NAT (*C*), malignant tumors and their NAT (*D*), and malignant and benign tumors (*E*). Points above the dashed lines represent significantly altered hydroxyproline peptides (two-sided *t* test, *p* value < 0.05, *p* values were adjusted by permutation-based FDR for multiple comparisons). Significantly downregulated hydroxyproline peptides are shown in *blue* (fold change < −1.5), and upregulated ones are shown in *pink* (fold change >1.5).

To ensure reproducible and unbiased analysis, we used four different groups, including six pairs of normal and PDAC tumors and six pairs of normal and benign pancreatic tumors, and the MS data were acquired in technical replicates as well. Principal component analysis (PCA) plot shows that the malignant group and benign group are well separated, supporting our purity classification (Fig. 4*A*). Then we performed hierarchical clustering analysis of all quantified proline hydroxylation sites to explore their profiles at different groups (Fig. 4*B*). The heatmap illustrates column-wise clustering of biological replicates in either malignant and its NAT group or benign and its NAT group, suggesting larger intergroup differences than intragroup variations. Samples from benign and malignant tumors and their NAT, on the other hand, are mixed, which suggests that the hydroxyproline alteration in tumors and their NAT is relatively minor. This also demonstrates the capability of our methods for reliable quantitative analysis of protein proline hydroxylation. In addition, dramatic alterations between benign tumor and malignant tumor groups are observed, indicating different patterns between these types of tumors.

For better pairwise comparison between groups, we performed paired or unpaired Student’s *t* test of combinations and graphically displayed the results using volcano plots (Fig. 4, *C*–*E*). Colored dots refer to significantly changed hydroxyproline peptides (*p* value < 0.05) identified with a fold change >1.5. As shown, 4, 39 and 197 significantly changed hydroxyproline peptides were identified in benign tumor and its NAT group, malignant tumor and its NAT group, and malignant and benign group, respectively (supplemental Table S4). These results indicate more abnormal proline hydroxylation occurs during PDAC progression and many of them exhibited higher expression levels in the malignant group. For instance, proline hydroxylation on some collagen proteins (*e.g.*, COL6A2) was upregulated in the malignant tumor group. COL6A2 is a part of the collagen VI family, which forms a key component of the ECM, providing structural support and involves cell adhesion and proliferation (43). A higher proline hydroxylation on these proteins may introduce structural changes in the protein that influence tumor angiogenesis, progression, and metastasis. Additionally, we also observed overexpression of proline hydroxylation on a few metabolic proteins such as glucose-6-phosphate isomerase (G6PI), which are associated with the metabolic demands and aggressive nature of PDAC cells (44, 45). Collectively, our results suggest that proline hydroxylation is altered and dysregulated in PDAC compared to controls, indicating that this modification may play a crucial role in pancreatic cancer pathology. This dysregulation highlights the potential of hydroxyproline sites could serve as biomarkers and further investigation is required to establish their potential roles in cancer development.

### Gene Ontology Analysis of Significantly Changed Hydroxyproline Peptides in PDAC and Benign Tumor Samples

**Fig. 5:**
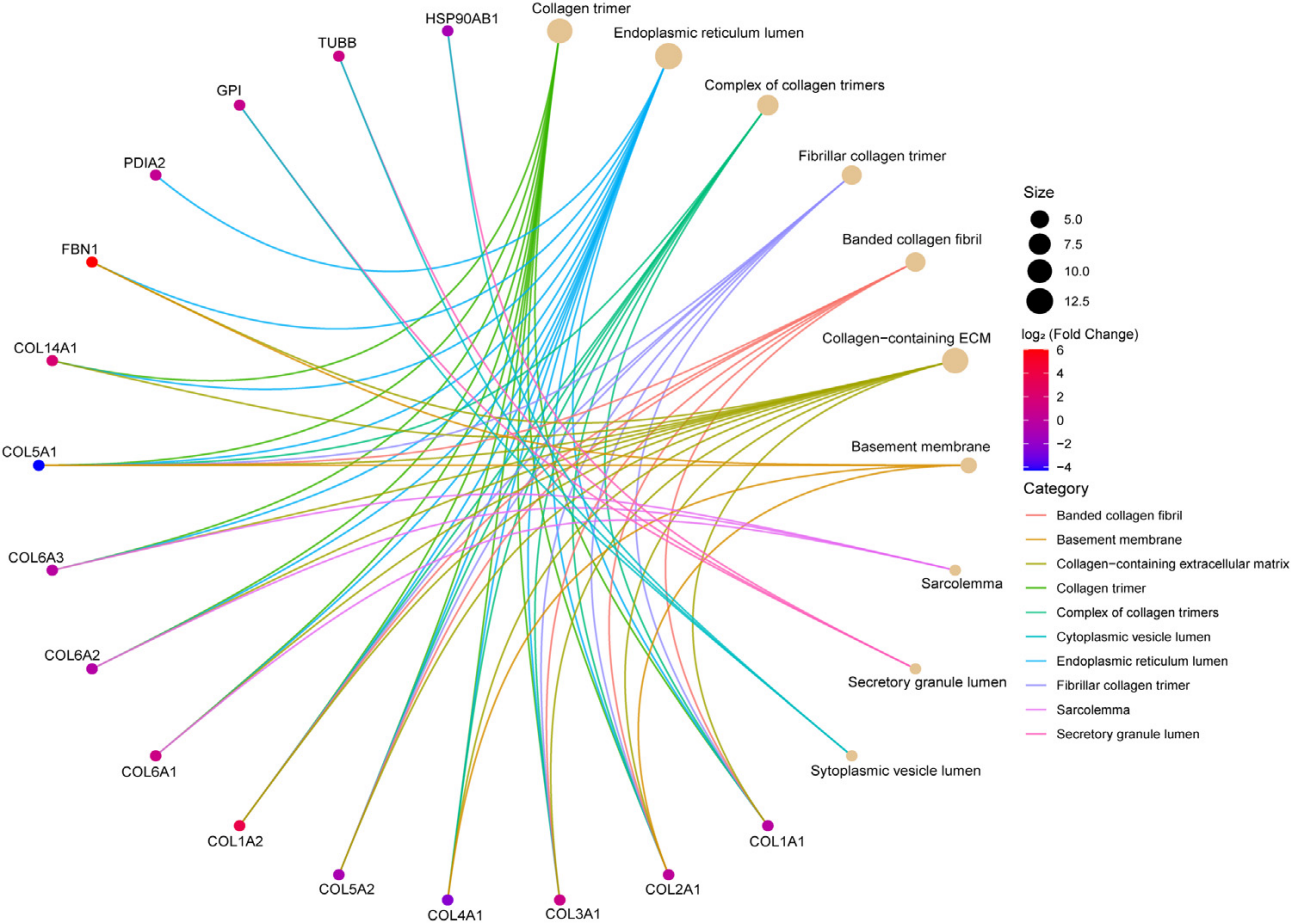
**Genenetwork plot of significantly changed hydroxyproline peptides in benign and malignant pancreatic tumors**. Plots were constructed using an overrepresentation analysis using the GO cellular component function database. The dot size of each network category is scaled by the number of overlapping hydroxyproline proteins within the category. The top 10 results are shown with a filtered FDR score of 0.05.

We constructed gene network plots with the significantly changed hydroxyproline peptides in PDAC and benign pancreatic tumors between using the GO cellular component function database (Fig. 5). Several categories were enriched, including collagen trimer, fibrillar collagen trimer, and collagen-containing ECM. Further, a KEGG pathway enrichment analysis of 197 significantly altered hydroxyproline peptides identified the "protein digestion and absorption" pathway as the top-scoring pathway, involving 11 subtypes of collagen proteins (COL3A1, COL6A1, COL6A3, COL14A1, COL5A2, COL1A2, COL4A1, COL5A1, COL2A1, COL6A2, and COL1A1) (supplemental Fig. S2). Collagen digestion is known to provide an amino acid supply, which is critical for cellular functions and cancer progression (46). Additionally, collagen degradation products, particularly those involving COL1A1 and COL1A2, can influence the epithelial-mesenchymal transition, a process associated with increased migratory and invasive capabilities of cancer cells (47). Therefore, the enrichment of this pathway suggests a significant role for the detected hydroxyproline peptides in PDAC progression. Taken together, our findings demonstrated that the significantly changed hydroxyproline peptides are potentially involved in the pathogenesis and progression of PDAC. These insights enhance our understanding of the molecular mechanisms driving PDAC and may inform future therapeutic strategies targeting the ECM and related pathways.

## Discussion

The current understanding of site-specific protein proline hydroxylation in PDAC is relatively sparse due to its complexity and limited high-throughput technologies available compared with other PTM studies. By utilizing an improved workflow that includes an optimized HILIC method and high-throughput quantification strategy with DiLeu isobaric tags, this study identified hundreds of hydroxyproline peptides from pancreatic cancer samples. To map the site-specific hydroxyproline forms in human pancreas, a multiplexed quantitative analysis was conducted using the pancreas samples pooled together from 10 healthy participants. This resulted in the identification of 194 hydroxyproline peptides from 59 proline-hydroxylated proteins, representing the first and the largest dataset of site-specific hydroxyproline forms from the human pancreas. Given that proline hydroxylation alterations have been implicated in cancer progression (10, 48), it would be highly valuable to obtain a description of the hydroxyproline form landscape of tissues in different groups in pancreatic cancer and make a comparison to those of healthy controls.

For biomarker discovery studies driven by proline hydroxylation, it is crucial to quantitatively demonstrate hydroxyproline forms comprehensively and determine their changes between disease and control states. Thus, a high-throughput quantitative hydroxyproline study was performed focusing on exploring the proline hydroxylation changes in patients using the paired normal PDAC and benign tumor tissues. This method not only enhanced multiplexing capacity but also minimized run-to-run variations and instrument time. Notably, we collected only five fractions and performed two technical replicates for each sample, yet still achieved a relatively comprehensive analysis. Protein coverage can be further improved by increasing the number of fractions, provided there are sufficient instrument resources. In total, a comparable number of hydroxyproline peptides were identified in PDAC tissue samples, with several hydroxyproline proteins/sites showing altered proline hydroxylation patterns. Here, some of the interesting targets have been selected for further discussion.

The collagen alpha-1(I) chain is a critical component of type I collagen, which is the most abundant collagen type in the ECM of various tissues, including the pancreas (49). Proline hydroxylation on COL1A1 is essential for the stability and function of collagen fibers, facilitating the formation of a stable triple helix structure necessary for maintaining the mechanical strength and integrity of the ECM (42). The observed upregulation of proline hydroxylation sites on proline 937 and 946 in benign tumors compared to control suggests an enhanced ECM stabilization mechanism that might be protective against tumor invasion and metastasis (supplemental Fig. S3*A*). In benign pancreatic tumors, the increased proline hydroxylation of COL1A1 could be indicative of a more robust ECM, which may serve as a physical barrier to tumor cell dissemination (50). In contrast, PDAC has more proline hydroxylation on collagen alpha-1(I) chain. The differential proline hydroxylation of COL1A1 in benign *versus* malignant tumors also implicates potential differences in the hypoxia signaling pathways between these tumor types (13). In benign tumors, higher levels of proline hydroxylation might be linked to a more effective hypoxia-inducible factor pathway regulation, ensuring adequate cellular responses to oxygen levels and preventing aggressive tumor behavior (13).

Collagen alpha-1(XII) is a key component of the ECM that plays a crucial role in modulating the structural integrity and mechanical properties of the tumor microenvironment (7, 10). Five hydroxyproline sites were found upregulated on collagen alpha-1(XII) in PDAC-only rather than benign tumors (supplemental Fig. S3*B*). This could be indicative of an adaptive response by the tumor to enhance its invasive capabilities. This is consistent with findings from studies that show how ECM components, including collagens, are dynamically regulated to support tumor growth and metastasis (42, 51). Furthermore, the heightened levels of COL12A1 might also be associated with the activation of specific signaling pathways that promote tumor survival and resistance to therapy (51). This highlights the complex interplay between ECM components and cellular signaling in driving the malignant phenotype of PDAC. Overall, our comprehensive quantitative analysis of hydroxyproline in the human pancreas provides valuable insights into the changes in this PTM during the progression of PDAC. We observed significant variations in the expression of many previously identified pancreatic tumor biomarkers across benign pancreatic tumors and PDAC. These findings could be important knowledge for studies of PTM-centric biomarker identification and their clinical applications. The differential proline hydroxylation of these proteins in benign *versus* malignant tumors highlights their promise as therapeutic targets. More importantly, this approach can be readily adopted by other researchers using various custom-developed or commercially available isobaric tags, offering a versatile and high-throughput strategy for analyzing protein proline hydroxylation. This strategy will greatly facilitate related studies to improve our understanding of proline hydroxylation under both physiological and pathological states.

## Data Availability

The LC-MS/MS raw data and annotated spectra have been deposited to the ProteomeXchange Consortium *via* the MassIVE partner repository with the accession number “MSV000095824”.

## Supplemental data

This article contains supplemental data.

## Conflict of interest

The authors declare that they have no conflicts of interest with the contents of this article.

## Supplementary Data

SI_HyP manuscript _2nd_round_Rev_Final_Clean
