# Supplementary material for: Proteome-Wide Investigation of Proline Hydroxylation in Pancreatic Ductal Adenocarcinoma Using DiLeu Isobaric Labeling Strategy
Source: Mol Cell Proteomics. 2025 Apr 9;24(7):100969. doi: 10.1016/j.mcpro.2025.100969 (PMC12275936; doi:10.1016/j.mcpro.2025.100969)
Supplement: SI_HyP manuscript _2nd_round_Rev_Final_Clean [file mmc1.docx]

Supporting Information

**Proteome-Wide Investigation of Proline Hydroxylation in Pancreatic Ductal Adenocarcinoma Using DiLeu Isobaric Labeling Strategy**

**Feixuan Wu,^1^ Dylan N. Tabang,^2,3^ Danqing Wang,^2^ Jon S. Odorico,^4^ Lingjun Li^1,2,5,6,7*^**

^1^ School of Pharmacy, University of Wisconsin-Madison, Madison, WI 53705, USA.

^2^ Department of Chemistry, University of Wisconsin-Madison, Madison, WI 53706, USA.

^3^ Department of Pathology, Boston Children’s Hospital & Harvard Medical School, Boston, MA 02115, USA

^4^ Department of Surgery, Division of Transplantation, School of Medicine and Public Health, University of Wisconsin-Madison, Madison, WI 53792, USA.

^5^ Biophysics Graduate Program, University of Wisconsin-Madison, WI 53706, USA.

^6^ Lachman Institute for Pharmaceutical Development, School of Pharmacy, University of Wisconsin-Madison, Madison, WI, 53705, USA

^7^ Wisconsin Center for NanoBioSystems, School of Pharmacy, University of Wisconsin-Madison, Madison, WI 53705, USA.

*For correspondence: Lingjun Li, [lingjun.li@wisc.edu](mailto:lingjun.li@wisc.edu)

**Table of Contents**

**Figure S1.** GRAVY scores throughout different methods.

**Figure S2.** KEGG pathway enrichment analysis.

**Figure S3.** Significantly changed proline hydroxylation sites for collagen alpha-1(I) (A) and collagen alpha-1(XII) (B) in tissues between benign tumors and PDAC. (VWFC: Von Willebrand factor type C; FCNC1: Fibrillar collagen NC1; VWFA: Von Willebrand factor type A)

**Table S1.** Healthy donor pancreas sample information.

**Table S2.** Paired pancreatic tumors and their NAT sample information.

**Table S3.** Hydroxyproline peptides identified from healthy donor pancreas (separate excel spreadsheet).

**Table S4.** Hydroxyproline peptides identified from pancreatic tumors and their NAT samples (separate excel spreadsheet).


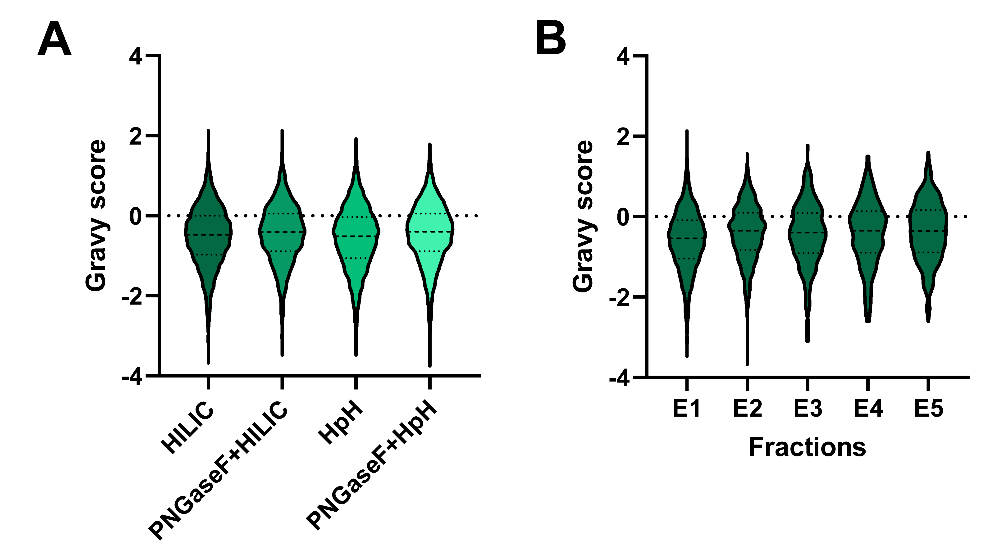


**Figure S1.** GRAVY scores from different methods (A) and HILIC method (B).


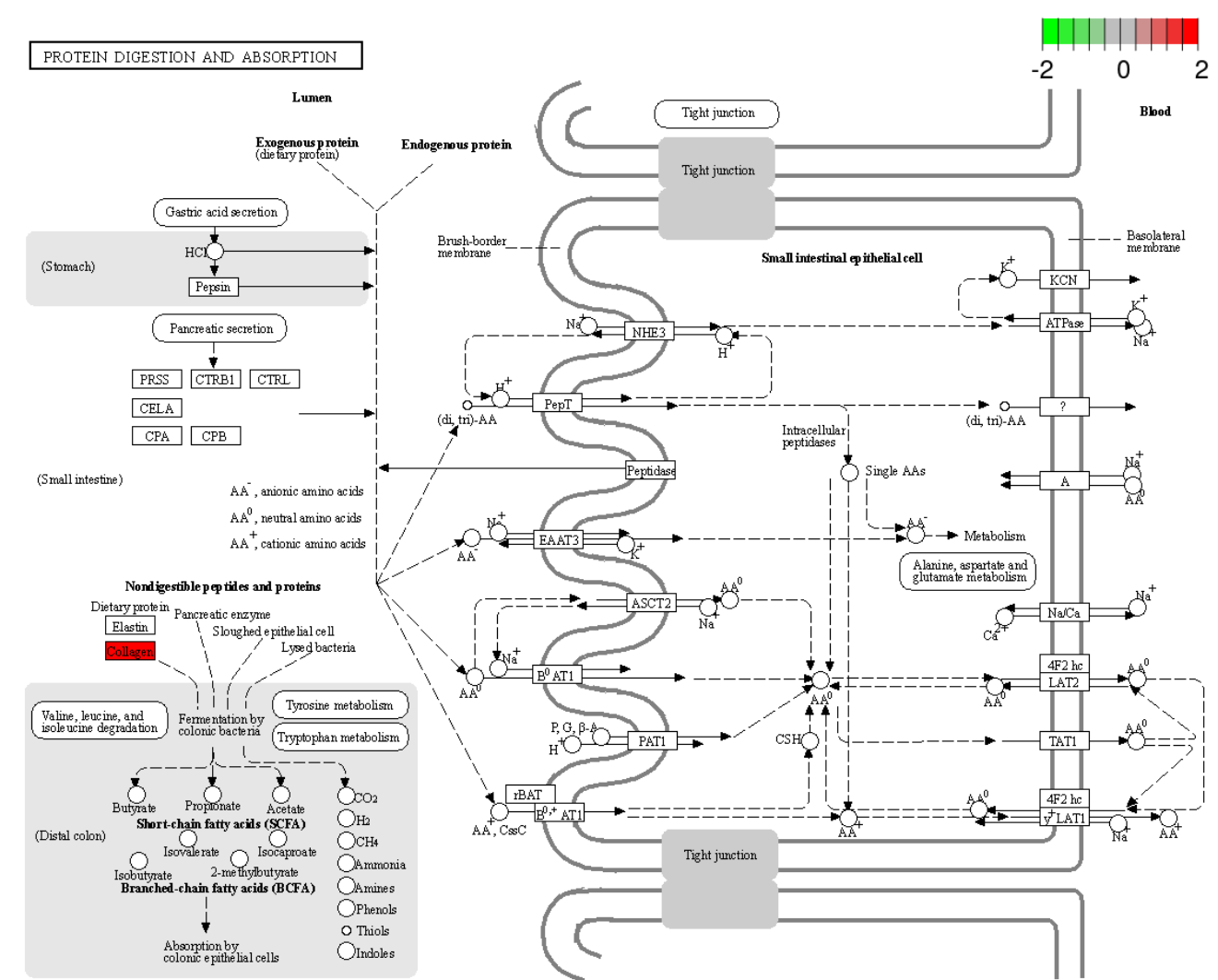


**Figure S2.** KEGG pathway enrichment analysis of 197 significantly changed hydroxyproline peptides revealed that the top-scoring pathway was “protein digestion and absorption”. Red highlight was detected in our present experiment.

**
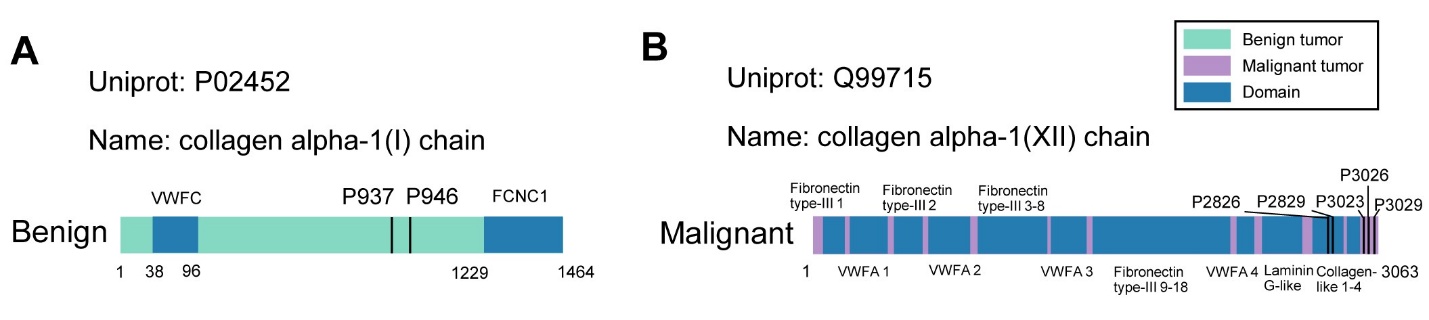
Figure S3.** Significantly changed proline hydroxylation sites for collagen alpha-1(I) (A), collagen alpha-1(XII) (B) in tissues from benign tumors and PDAC. (VWFC: Von Willebrand factor type C; FCNC1: Fibrillar collagen NC1; VWFA: Von Willebrand factor type A)

| **Number** | **Gender** | **Age (years)** | **Diagnosis** | **BMI** |
| --- | --- | --- | --- | --- |
| **1** | **Female** | **48** | **Not diabetic** | **20.7** |
| **2** | **Female** | **46** | **Not diabetic** | **24.7** |
| **3** | **Female** | **26** | **Not diabetic** | **20.1** |
| **4** | **Female** | **29** | **Not diabetic** | **34.4** |
| **5** | **Female** | **28** | **Not diabetic** | **27.1** |
| **6** | **Male** | **35** | **Not diabetic** | **23.1** |
| **7** | **Male** | **39** | **Not diabetic** | **22.2** |
| **8** | **Male** | **38** | **Not diabetic** | **23.6** |
| **9** | **Male** | **24** | **Not diabetic** | **22.5** |
| **10** | **Male** | **43** | **Not diabetic** | **29.3** |
| **Average** |  | **35.6±8.58** |  | **24.8±4.38** |

**Table S1.** Healthy donor pancreas samples information. Average is presented as mean ± standard deviation.

| **Number** | **Gender** | **Age (years)** | **Diagnosis** | **Stage** |
| --- | --- | --- | --- | --- |
| **1** | **Female** | **68** | **Malignant** | **II** |
| **2** | **Female** | **58** | **Malignant** | **II** |
| **3** | **Female** | **70** | **Malignant** | **II** |
| **4** | **Male** | **70** | **Malignant** | **IV** |
| **5** | **Male** | **62** | **Malignant** | **II** |
| **6** | **Male** | **77** | **Malignant** | **II** |
| **Average** |  | **67.5±6.69** |  |  |
| **7** | **Female** | **64** | **Benign** | **NA** |
| **8** | **Female** | **81** | **Benign** | **NA** |
| **9** | **Female** | **68** | **Benign** | **NA** |
| **10** | **Female** | **24** | **Benign** | **NA** |
| **11** | **Male** | **66** | **Benign** | **NA** |
| **12** | **Male** | **73** | **Benign** | **NA** |
| **Average** |  | **62.67±19.90** |  |  |

**Table S2.** Paired pancreatic tumors and their NAT samples information. Average is presented as mean ± standard deviation. NA means not available.
